# Supplementary material for: Prognostic Relevance of CD4+, CD8+ and FOXP3+ TILs in Oral Squamous Cell Carcinoma and Correlations with PD-L1 and Cancer Stem Cell Markers
Source: Biomedicines. 2021 Jun 8;9(6):653. doi: 10.3390/biomedicines9060653 (PMC8227658; doi:10.3390/biomedicines9060653)
Supplement: Supplementary file 1 [file biomedicines-09-00653-s001.zip › biomedicines-1202756-supplementary.pdf]

Supplementary material

Supplementary Figure S1. Stromal and tumoral distribution of $CD8^+$, $CD4^+$ and $FOXP3^+$ T cell subsets in OSCC patients. Whiskers indicate variability outside the 75 and 25 percentiles. The Y axis represents the number of infiltrating T cells. Circles represent outliers and asterisks extreme values.

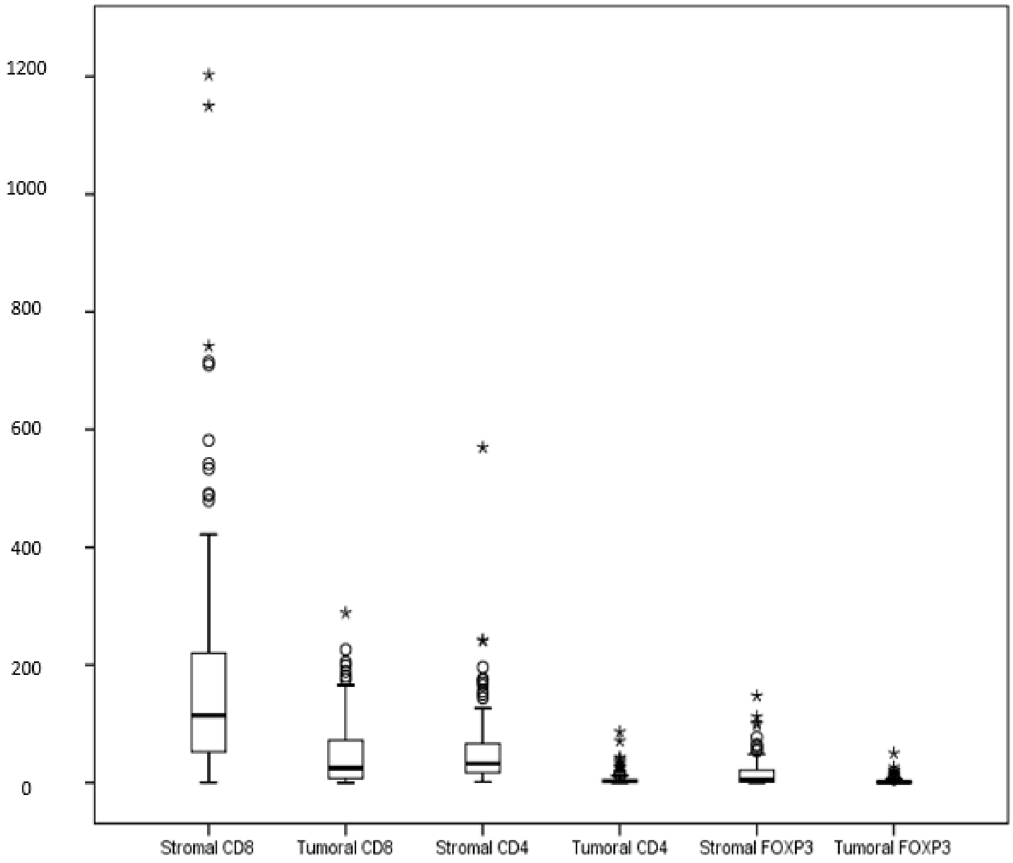

Supplementary Table S1. Correlations between the mean numbers of CD4, CD8 and FOXP3 TIL infiltration in the tumor nests and surrounding stroma. The Spearman's Rho coefficients and the corresponding *p* values are shown.

**Factor**	*Tumoral CD4 (mean)*	*Stromal CD8 (mean)*	*Tumoral CD8 (mean)*	*Stromal FOXP3 (mean)*	*Tumoral FOXP3 (mean)*
Stromal CD4 (mean)	0.693<br>< 0.001	0.693<br>< 0.001	0.401<br>< 0.001	0.365<br>< 0.001	0.262<br>0.003
Tumoral CD4 (mean)		0.467<br>< 0.001	0.418<br>< 0.001	0.252<br>0.005	0.224<br>0.013
Stromal CD8 (mean)			0.515<br>< 0.001	0.320<br>< 0.001	0.265<br>0.003
Tumoral CD8 (mean)				0.094<br>0.299	0.259<br>0.004
Stromal FOXP3 (mean)					0.817<br>< 0.001

Supplementary Table S2. Associations between stromal and tumoral CD8/FOXP3 ratios and clinicopathological parameters in the cohort of 125 OSCC patients.

Variable	Stromal CD8/FOXP3 ratio Mean (SD)	*p*	Tumoral CD8/FOXP3 ratio Mean (SD)	*p*
Age (years)				
< 65	26.74 (41.18)	0.001	14.80 (20.47)	0.001
≥ 65	80.96 (157.80)		55.79 (84.12)	
Gender				
Female	54.87 (133.00)	0.52	33.07 (70.27)	0.67
Male	45.45 (92.93)		28.43 (49.00)	
Tobacco				
No	74.12 (150.44)	0.10	51.66 (87.01)	0.01
Yes	35.68 (76.03)		18.59 (25.57)	
Alcohol				
consumption	60.98 (130.48)	0.11	47.90 (78.43)	0.005
No	37.81 (82.87)		15.50 (21.53)	
Yes				
T classification				
T1 + 2	36.02 (56.97)	0.76	27.05 (53.78)	0.11
T3 + 4	76.87 (172.71)		36.75 (63.93)	
N classification				
N0	58.92 (134.09)	0.73	34.79 (68.05)	0.90
N+	32.60 (38.09)		21.56 (26.38)	
Stage				
I + II	35.16 (63.75)	0.40	29.67 (63.06)	0.12
III + IV	59.15 (132.01)		30.35 (51.72)	
Grade				
Well	49.62 (102.66)	0.12	29.21 (55.88)	0.87
Moderate + Poor	47.21 (118.14)		31.29 (59.19)	
Site				
Tongue	22.80 (32.87)	0.16	21.40 (34.04)	0.35
Rest	65.76 (134.23)		36.05 (68.13)	
Recurrence				
No	48.00 (106.23)	0.08	32.54 (68.62)	0.11
Yes	49.77 (111.83)		25.81 (28.03)	
Second primary				
tumor	45.64 (110.95)	0.37	29.61 (58.50)	0.76
No	63.87 (93.98)		32.20 (49.80)	
Yes				

All *p*-values were calculated using the U Mann-Whitney test.

A

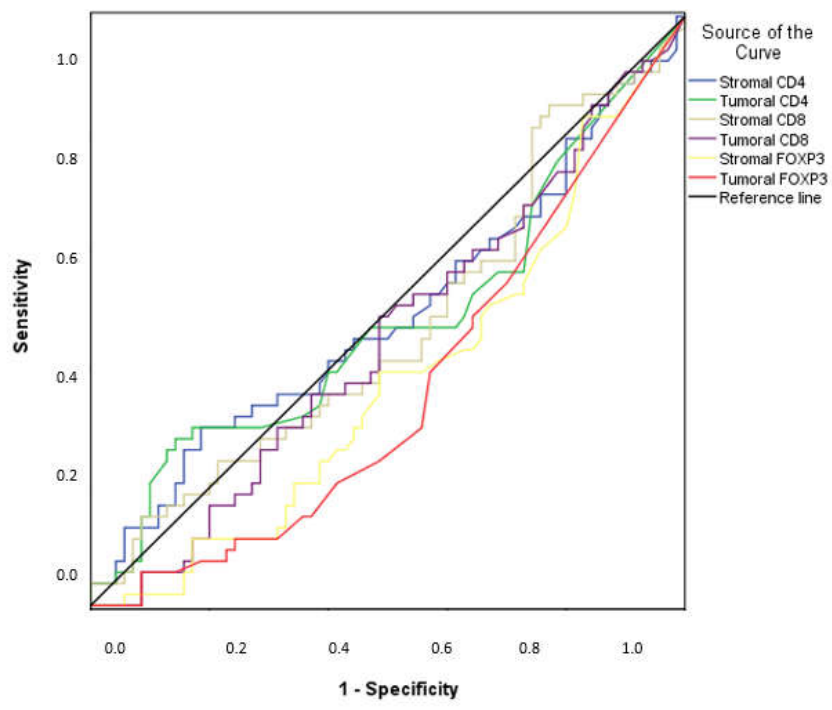

B

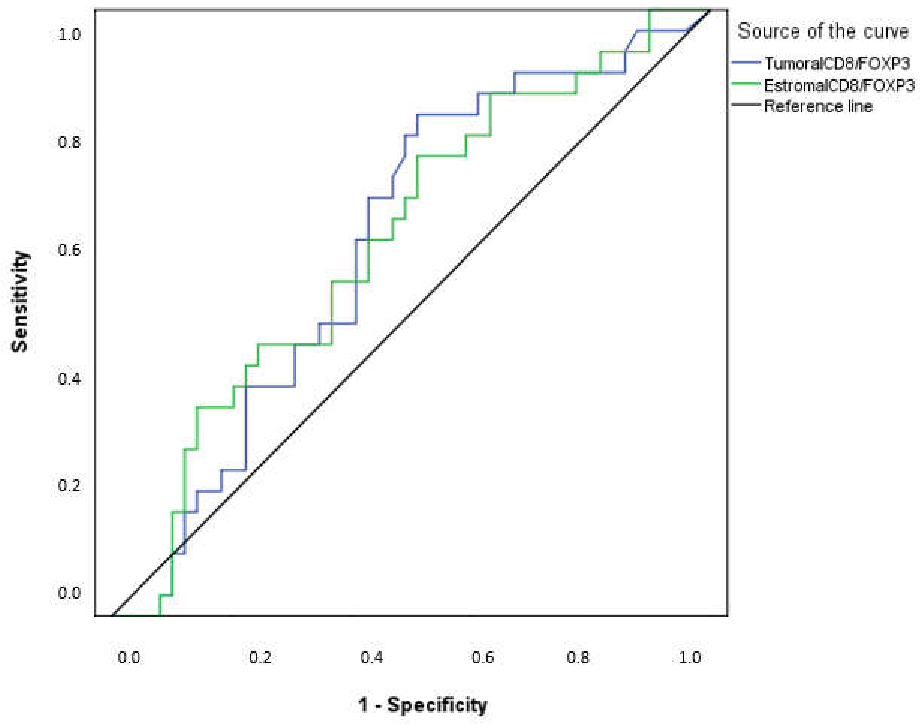

Supplementary Figure S2. ROC curves of stromal/tumoral CD4, CD8, and FOXP3 (A) and stromal/tumoral CD8/FOXP3 ratio (B).

Supplementary Table S3. DSS predictive accuracy of CD4, CD8 and FOXP3 infiltrating TILs.

**Predictive factor**	**AUC**	**SE**	***p***	**95% CI**
Stromal CD8	0.472	0.053	0.594	0.368 – 0.576
Tumoral CD8	0.455	0.052	0.391	0.352 – 0.557
Stromal CD4	0.494	0.054	0.902	0.388 – 0.599
Tumoral CD4	0.484	0.054	0.755	0.377 – 0.590
Stromal CD8/CD4 ratio	0.460	0.053	0.450	0.356 – 0.564
Tumoral CD8/CD4 ratio	0.462	0.053	0.476	0.359 – 0.566
Stromal FOXP3	0.384	0.051	0.028	0.285 – 0.483
Tumoral FOXP3	0.368	0.050	0.012	0.270 – 0.466
Stromal CD8/FOXP3 ratio	0.621	0.065	0.076	0.494 – 0.748
Tumoral CD8/FOXP3 ratio	0.616	0.065	0.089	0.489 – 0.742

Supplementary Table S4. Stratified univariate Kaplan-Meier analysis of stromal and tumoral CD8+ and FOXP3+ TILs with clinicopathological variables in 125 OSCC patients. Median values were used as cut-off points. No correlations were found with stromal and tumoral CD4.

**Parameters**	**Number**	**Censored patients (%)**	**Cancer-free survival time (95% CI)**	***p***
Age, stromal CD8+				
< 65 years				
• ≤ 118	38	17 (45)	106.58 (74.56 – 138.60)	0.01
• > 118	39	29 (74)	175.97 (147.19 – 204.76)	
≥ 65 years				
• ≤ 118	25	15 (60)	82.36 (58.26 – 106.47)	0.89
• > 118	23	11 (48)	93.20 (66.04 – 120.36)	
Age, tumoral FOXP3+				
< 65 years				
• ≤ 0.666	30	13 (43)	107.69 (71.99 – 143.39)	0.02
• > 0.666	47	33 (70)	166.16 (138.40 – 193.91)	
≥ 65 years				
• ≤ 0.666	23	12 (52)	78.05 (50.08 – 106.02)	0.47
• > 0.666	25	14 (56)	93.20 (69.40 – 124.63)	
Gender, stromal CD8+				
Female				
• ≤ 118	23	14 (61)	135.66 (92.84 – 178.49)	0.81
• > 118	20	11 (55)	113.00 (84.50 – 141.49)	
Male				
• ≤ 118	40	18 (52)	84.07 (61.26 – 106.89)	0.02
• > 118	42	29 (56)	161.57 (131.04 – 192.10)	
Gender, stromal FOXP3+				
Female				
• ≤ 5.666	19	9 (47)	71.52 (44.59 – 98.45)	0.03
• > 5.666	24	16 (67)	155.47 (118.21 – 192.73)	

Male				
• ≤ 5.666	41	20 (49)	100.56 (75.60 – 125.52)	0.28
• > 5.666	41	27 (66)	149.26 (115.52 – 183.00)	
Gender, tumoral FOXP3⁺				
Female				
• ≤ 0.666	20	9 (45)	93.61 (48.10 – 139.13)	0.01
• > 0.666	23	16 (70)	141.86 (112.04 – 171.8)	
Male				
• ≤ 0.666	33	16 (48)	99.79 (71.88 – 127.71)	0.28
• > 0.666	49	31 (63)	145.57 (115.14 – 176.00)	
Gender, stromal CD8/FOXP3⁺				
Female	18	14 (78)	178.76 (141.20 – 216.32)	0.009
• ≤ 10.9853	19	7 (37)	76.45 (48.82 – 104.09)	
• > 10.9853				
Male	25	16 (64)	145.09 (101.67 – 188.50)	0.63
• ≤ 10.9853	44	25 (57)	107.18 (83.93 – 130.43)	
• > 10.9853				
Tobacco, stromal CD8⁺				
No				
• ≤ 118	17	11 (65)	89.20 (57.66 – 120.74)	0.92
• > 118	24	13 (54)	106.56 (78.23 – 134.89)	
Yes				
• ≤ 118	46	21 (46)	103.66 (73.71 – 133.60)	0.02
• > 118	38	27 (71)	165.05 (133.02 – 197.09)	
Tobacco, stromal FOXP3⁺				
No				
• ≤ 5.666	21	12 (57)	103.91 (71.07 – 136.74)	0.78
• > 5.666	20	12 (60)	155.47 (75.17 – 137.00)	
Yes				
• ≤ 5.666	39	17 (44)	90.67 (75.60 – 125.52)	0.02
• > 5.666	45	31 (69)	159.52 (129.59 – 189.45)	
Tobacco, tumoral FOXP3⁺				

No<br>• ≤ 0.666<br>• > 0.666<br><br>Yes<br>• ≤ 0.666<br>• > 0.666	 17<br>24<br><br><br>36<br>48	 10 (59)<br>14 (58)<br><br><br>15 (42)<br>33 (69)	 103.25 (66.07 – 140.44)<br>109.26 (79.72 – 138.79)<br><br><br>100.20 (67.33 – 133.07)<br>159.12 (130.00 – 188.24)	 0.74<br><br><br><br>0.01
Alcohol, stromal CD8+<br><br>No<br>• ≤ 118<br>• > 118<br><br>Yes<br>• ≤ 118<br>• > 118	 <br><br>25<br>31<br><br><br>38<br>31	 <br><br>14 (56)<br>17 (55)<br><br><br>18 (47)<br>23 (74)	 <br><br>122.59 (80.18 – 165.00)<br>105.97 (80.51 – 131.43)<br><br><br>96.37 (69.78 – 122.96)<br>171.48 (137.03 – 205.94)	 <br><br>0.65<br><br><br><br>0.02
Alcohol, stromal FOXP3+<br><br>No<br>• ≤ 5.666<br>• > 5.666<br><br>Yes<br>• ≤ 5.666<br>• > 5.666	 <br><br>27<br>29<br><br><br>33<br>36	 <br><br>14 (52)<br>17 (59)<br><br><br>15 (45)<br>26 (72)	 <br><br>95.55 (66.73 – 124.37)<br>135.95 (98.90 – 173.00)<br><br><br>93.79 (65.88 – 121.70)<br>165.31 (132.39 – 198.23)	 <br><br>0.55<br><br><br><br>0.02
Alcohol, tumoral FOXP3+<br><br>No<br>• ≤ 0.666<br>• > 0.666<br><br>Yes<br>• ≤ 0.666<br>• > 0.666	 <br><br>25<br>31<br><br><br>28<br>41	 <br><br>13 (52)<br>18 (58)<br><br><br>12 (43)<br>29 (71)	 <br><br>113.43 (72.02 – 154.85)<br>108.18 (81.99 – 134.36)<br><br><br>89.92 (59.68 – 120.15)<br>163.06 (132.05 – 194.08)	 <br><br>0.45<br><br><br><br>0.01
Alcohol, stromal CD8/FOXP3+<br><br>No<br>• ≤ 10.9853<br>• > 10.9853	 <br><br>16<br>34	 <br><br>9 (56)<br>18 (53)	 <br><br>135.15 (86.44 – 183.87)<br>97.16 (71.57 – 122.76)	 <br><br>0.68

Yes				
• ≤ 10.9853	27	21 (78)	177.29 (140.49 – 214.09)	0.03
• > 10.9853	29	14 (48)	98.76 (71.74 – 125.78)	
Tumor site, stromal FOXP3+				
Tongue				
• ≤ 5.666	20	8 (40)	73.61 (37.53 – 109.68)	0.03
• > 5.666	31	20 (65)	147.75 (110.08 – 185.42)	
Other				
• ≤ 5.666	40	21 (53)	101.60 (78.64 – 124.56)	0.17
• > 5.666	34	23 (68)	135.44 (108.80 – 162.08)	
Tumor site, stromal CD8/FOXP3+				
Tongue				
• ≤ 10.9853	17	13 (77)	180.37 (138.19 – 222.53)	0.01
• > 10.9853	25	10 (40)	75.44 (45.34 – 105.54)	
Other				
• ≤ 10.9853	26	17 (65)	129.64 (97.54 – 161.74)	0.58
• > 10.9853	38	22 (58)	109.58 (87.11 – 132.04)	
N classification, stromal CD8+				
N0				
• ≤ 118	39	24 (62)	118.84 (91.70 – 145.99)	0.37
• > 118	37	25 (68)	133.75 (110.74 – 156.76)	
N+				
• ≤ 118	24	8 (33)	71.93 (33.70 – 110.17)	0.03
• > 118	25	15 (60)	141.96 (100.24 – 183.68)	
Grade, stromal CD8+				
Well differentiated				
• ≤ 118	38	17 (45)	97.72 (63.86 – 131.58)	0.03
• > 118	42	27 (64)	151.03 (119.76 – 182.30)	
Moderate-poorly				
differentiated	25	15 (60)	112.06 (75.92 – 148.21)	0.57
• ≤ 118	20	13 (65)	117.90 (85.90 – 149.90)	
• > 118				
Grade, stromal CD8/FOXP3+				

Well differentiated<br>• ≤ 10.9853<br>• > 10.9853<br><br>Moderate-poorly differentiated<br>• ≤ 10.9853<br>• > 10.9853	 24<br>43<br><br><br>19<br>20	 17 (71)<br>19 (44)<br><br><br>13 (68)<br>13 (65)	 180.37 (138.19 – 222.53)<br>75.44 (45.34 – 105.54)<br><br><br>131.76 (93.63 – 169.90)<br>111.49 (77.60 – 145.38)	 0.01<br><br><br><br>0.85
Radiotherapy, stromal $CD8^+$<br><br>No<br>• ≤ 118<br>• > 118<br><br>Yes<br>• ≤ 118<br>• > 118	 <br><br>27<br>23<br><br><br>36<br>39	 <br><br>23 (85)<br>21 (91)<br><br><br>9 (25)<br>19 (49)	 <br><br>163.76 (139.24 – 188.28)<br>133.75 (145.45 – 188.54)<br><br><br>64.86 (38.00 – 91.73)<br>123.34 (91.03 – 155.64)	 <br><br>0.50<br><br><br><br>0.01
Radiotherapy, tumoral $FOXP3^+$<br><br>No<br>• ≤ 0.666<br>• > 0.666<br><br>Yes<br>• ≤ 0.666<br>• > 0.666	 <br><br>21<br>29<br><br><br>32<br>43	 <br><br>17 (81)<br>27 (93)<br><br><br>8 (25)<br>20 (47)	 <br><br>142.44 (107.90 – 176.98)<br>178.24 (161.19 – 195.29)<br><br><br>70.28 (39.90 – 100.66)<br>115.05 (84.23 – 145.87)	 <br><br>0.15<br><br><br><br>0.04
PD-L1, stromal $FOXP3^+$<br><br>Negative (≤ 10% tumor cells)<br>• ≤ 5.666<br>• > 5.666<br><br>Positive (> 10% tumor cells)<br>• ≤ 5.666<br>• > 5.666	 <br><br>50<br>54<br><br><br>8<br>10	 <br><br>25 (50)<br>38 (70)<br><br><br>3 (38)<br>4 (40)	 <br><br>99.36 (76.40 – 122.32)<br>163.75 (137.04 – 190.45)<br><br><br>52.28 (13.99 – 90.57)<br>75.60 (31.13 – 120.06)	 <br><br>0.02<br><br><br><br>0.66
PD-L1, tumoral $FOXP3^+$<br><br>Negative (≤ 10% tumor cells)<br>• ≤ 0.666<br>• > 0.666	 <br><br>44<br>60	 <br><br>21 (48)<br>42 (70)	 <br><br>107.44 (76.61 – 138.27)<br>163.68 (138.49 – 188.87)	 <br><br>0.01

Positive (> 10% tumor cells)<br>• ≤ 0.666<br>• > 0.666	 6<br>12	 2 (33)<br>5 (42)	 41.80 (1.00 – 82.60)<br>76.75 (37.37 – 116.12)	 0.37
SOX2, stromal CD8^{+}<br><br>Negative<br>• ≤ 118<br>• > 118<br><br>Positive<br>• ≤ 118<br>• > 118	 <br><br>34<br>38<br><br><br>28<br>21	 <br><br>14 (41)<br>23 (61)<br><br><br>18 (64)<br>14 (67)	 <br><br>67.04 (46.25 – 87.82)<br>138.78 (104.52 – 173.05)<br><br><br>140.19 (101.09 – 179.29)<br>127.15 (99.33 – 154.97)	 <br><br>0.03<br><br><br><br>0.64
NANOG, stromal CD8^{+}<br><br>Negative<br>• ≤ 118<br>• > 118<br><br>Positive<br>• ≤ 118<br>• > 118	 <br><br>38<br>45<br><br><br>24<br>15	 <br><br>17 (45)<br>28 (62)<br><br><br>14 (58)<br>10 (67)	 <br><br>97.72 (64.16 – 131.29)<br>146.36 (115.80 – 176.91)<br><br><br>102.06 (76.12 – 127.99)<br>124.56 (87.43 – 161.70)	 <br><br>0.04<br><br><br><br>0.67

p values were estimated using the log-rank test.

Supplementary Table S5. Multivariate Cox regression of disease-specific survival for clinical variables and TILs infiltration.

Variable	*p*	Hazard Ratio	95% CI
Stage (I + II vs. III + IV)	0.01	2.195	1.170 – 4.119
Stromal CD8/FOXP3 ratio (≤10.9853 vs. > 10.9853)	0.03	2.039	1.059 – 3.925
